# 

**Published:** 1880-01

**Authors:** 


### CONTENTS VOL. XXXIV.

A Course of Lectures on Operative Dental Surgery and Therapeutics ..... 119
A New Antiseptic ..... 140
Address of Ohio College of Dental Surgery ..... 153
Address of Mississippi Valley Dental Association ..... 165
A Word of Caution in the Administration of Acid Medicines ..... 187
A Case of Hare-Lip ..... 188
Address at the Commencement of the Dental College at Ann Arbor ..... 201
Afternoon Session, March 3d, 1880 ..... 218
A Directory of Dentists ..... 280
A Powerful Disinfectant ..... 283
American Dental Association ..... 291
A New Medical Journal ..... 292
Address ..... 304
An Important Movement ..... 327
A New Appliance ..... 335
Articulating Artificial Teeth ..... 356
Absence ..... 470
A Suggestion ..... 508
A New "Special" Pattern Tooth Brush ..... 557
An American Elephant ..... 548
A Short Paper on Nervous Reflex Action ..... 532
Another Mastodon ..... 547
Bibliographical ..... 47
Baltimore College of Dental Surgery ..... 233
Bibliographical ....423, 336, 288, 286
Cancroid Stomatitis ..... 57
Clinical Reports ...185, 266, 320, 142
Chemical Action on Tooth Bone and Fillings ..... 256
California State Dental Association ..... 291
Connecticut Valley Dental Society ..... 289
Correction ..... 292
Cylinder Filling ..... 316
Correspondence .....420, 325
Call for a Mass Convention ..... 328
Cohesive vs Non-cohesive Gold ..... 349
Carlessness or Ignorance, Which ..... 378
Compensation for Dental Services ..... 464
Cure for the Tobacco Habit ..... 491
Children's Hats ..... 549
Clinic Reports ..... 554
Correspondence ..... 553
Discussions at the Ohio State Society ..... 219
Dr. S. S. White's Death ..... 43
Dental Education ..... 78
Dyspepsia and the Teeth ..... 170
Discussions of the Thirty-Sixth Annual Meeting of the Mississippi Valley Dental Association ..... 172
Dental Department of Vanderbilt University ..... 239
Dental Department of University of Michigan ..... 239
Dental Education ..... 293
Dennett's Dental Naboli ..... 331
Diagnosis of Malignant Tumors of the Upper Jaw in Youth ..... 425
Diversion ..... 468
"Dental Ethics — Fee Bills, Tooth Powders, and Mouth Washes, their Uses and Abuses" ..... 471
Dental Education in Hungary ..... 499
Editorial .....94, 147, 195, 234, 280, 327, 378, 420
Extraction of First Molars ..... 129
Effects of tobacco on the Eyes ..... 140
Excursion ..... 337
Engagement Book ..... 470
Extracts from an Address ..... 484
Examining Board ..... 512
Easy Test for Arsenic in Fabrics ..... 548
Fate of the Discoverers of Anæsthesia ..... 552
Fur on the Tongue ..... 501
Graduates in Dentistry, 1880 ..... 233
General Care of the Mouth for Patients Under Twenty Years of Age ..... 432
How to Succeed in Our Profession ..... 186
How to Test Chloroform ..... 193

### INDEX.

How our School Makes Doctors 506
How to Distinguish Oleomargarine 196
Indiana State Dental Society 541
Illinois State Dental Society 244
"In Chains" 421
Miscellaneous Notes 41, 91, 144, 242
Meeting 290
Minutes of the Kentucky State Dental Society 369
Materia Medica and Therapeutics 438
Methods of Preserving the Brain 504
Northern Ohio Dental Association 198
New York College of Dentistry 238
New Substitute for Rubber 546
On the Value of Retaining the Roots of Teeth after the loss of their Crowns 117
One Step Ahead 147
Ohio College of Dental Surgery 234
On the Importance of the Teeth in Reference to the Health 249
Of Individual Importance to Every One Interested in Dentistry 284
Obituary 336, 423, 424, 292
Observations on the Secretion of Saliva in a Case of Parotid Fistula 503
On the Absolute Invisibility of Atoms and Molecules 550
On Vaccination 548
Our Duty 542
Obituary 558
Professional Ethics 70
Pennsylvania College of Dental Surgery 235
Philadelphia Dental College 237
Proceedings of the Twenty-First Annual Meeting of the Northern Ohio Dental Association 269
Pathology and Semiology 310
Physiology of the Salivary Glands 339
Proceedings of the Twentieth Annual Meeting of the American Dental Association 395
Preparation for Medical and Dental Study 459
Physicians' Visiting List 557
Report of the Committee of Dental Chemistry 66
Removal of Parotid Tumor.—Death from Cellulitus 116
Report of the Kentucky State Dental Association 359
Relations of the Profession and the Public 381
Report of the discussions before the Kentucky State Dental Assoc'n 444
Salivary Calculus and its Concomitants 7
Scientific News 89, 143, 241, 321
Sarcoma of Jaw—Death 192
Sun-Spots and Meteorology 263
Synopsis of an Introductory Lecture to a course of Dental Mechanics 496
Some Points in the Natural History of Caries of the Teeth, and the Value of Fillings for its Arrest 515
Saliva—Its Characteristics in Health and Disease 526
Tobacco 21
Thirty-Sixth Annual Meeting, Mississippi Valley Association of Dental Surgeons 133
The New Anæsthetic 190
Tardy Eruption of the Teeth 318
Treatment of Deciduous Teeth 390
The History of the Philadelphia Fraudulent Medical Colleges 476
The Retrospective View of Dentistry 481
The Population of the World 498
Swarming Extraordinary 547
The Apostles Creed 544
The Voice 545
The Rubber Patents 552
The Scientific American 556
What was the Cause of the Death of Geo. A. Gardner 15
Women who Smoke 189
Wickersheimer's Preservative Fluid 191
What Do You Do It For 299
What Kills 443

### CONTENTS.

COMMUNICATIONS.

Salivary Calculus and its Concomitants ........................................ 7
What was the cause of the Death of Geo. A. Gardner? .................. 15
Discussions at the Ohio State Dental Society ................................ 21

SCIENTIFIC NEWS.

Edison's Electric Lamp ........................................................ 36

Miscellaneous Notes ........................................................ 41

Dr. S. S. White's Death ........................................................ 43

EDITORIAL.

Bibliographical ........................................................ 47

---

### THE DENTAL REGISTER,

A MONTHLY MAGAZINE DEVOTED TO THE INTERESTS OF

**THE DENTAL PROFESSION.**

*TERMS REDUCED TO $2.50 PER YEAR. SINGLE COPIES 25C.*

EDITED BY PROF. J. TAFT, D.D.S.

AND

*Published by SPENCER, CROCKER & CO., Ohio Dental Depot.*

The volume commences in January and closes in December.

The REGISTER being issued monthly is always fresh, therefore Indispensable to the practitioner who desires to keep posted up with the new inventions and improvements which are daily developed. Neither expense nor effort will be spared to give value and variety to its contents. Articles will be illustrated with well executed engravings whenever they will add to the interest or assist to explanation.

Its extensive circulation and frequency of issue make it one of the BEST DENTAL ADVERTISING MEDIUMS in the United States.

All subscriptions must begin with January or July.

Local or special notices, five lines or less, will be inserted for$3 each insertion; over five lines, 50 cts. per line.

All advertising bills payable in advance, or at furthest, after the issue of the first number containing the advertisement. All transient advertisements to be paid for in advance.

☞CONTRIBUTIONS SOLICITED.

Exclusively Editorial Communications should be addressed to Editor.

The latest number of the REGISTER may be ordered with or without other goods at any time, and all letters should be addressed to

***SPENCER, CROCKER & CO., Ohio Dental Depot, Cincinnati, O.***

